# Effects on Children’s Physical and Mental Well-Being of a Physical-Activity-Based School Intervention Program: A Randomized Study

**DOI:** 10.3390/ijerph20031927

**Published:** 2023-01-20

**Authors:** Santo Marsigliante, Manuel Gómez-López, Antonella Muscella

**Affiliations:** 1 Department of Biological and Environmental Sciences and Technologies (Di.S.Te.B.A.), University of Salento, 73100 Lecce, Italy; 2 Department of Physical Activity, Sport Faculty of Sports Science, University of Murcia, 30720 Murcia, Spain

**Keywords:** school-based intervention, children relative body fat mass, overweight, BMI, waist circumference, standing long jump test, Ruffier test, sit and reach test, concentration test, well-being

## Abstract

This study aimed to evaluate the effectiveness of physically active breaks of a total duration of 10 min a day, introduced during curricular lessons, together with a 10 min physical activity intervention during the daily school recess period on obesity prevention, fitness, cognitive function, and psychological well-being in school-aged children. A sample of 310 children (139 boys vs. 171 girls), aged between 8 and 10 years (9.82 ± 0.51), was selected. Our strategy was implemented over a 6-month period and the participants were randomly assigned to either the intervention group (*n* = 157) or the non-intervention (control) group (*n* =153). In the intervention group, a significant decrease (*p* < 0.05) in body mass index, waist circumference, waist–height ratio, and relative body fat mass was achieved after the intervention (T1) compared to the values measured before intervention (T0); in the control group, no differences emerged between T0 and T1 for any of the parameters considered. We found a significant increase in the intervention group in standing long jump, Ruffier, and sit and reach test scores (*p* < 0.001 for all). At T0, cognitive test scores did not differ between the girls and boys or between the intervention and control groups; instead at T1, significant differences were observed in the two groups regarding the total number of responses and the concentration performance scores (*p* < 0.001). Consistently, in the intervention group, well-being levels significantly increased between T0 and T1 (*p* < 0.001). Finally, the intervention had significant effects on the children regardless of gender. We may therefore conclude that schools should create more opportunities for teachers and students to introduce intervention strategies to promote regular PA during school recess.

## 1. Introduction

In recent years, there has been a change in the lifestyles of various age groups, especially in late childhood. Today’s children, in fact, lead an increasingly sedentary lifestyle which encompasses the time spent by playing video games, using computers/smartphones, and watching television [1]. This lifestyle leads them to neglect the physical activity (PA) typical of this period of development [2]; this has negative implications in that PA has several positive effects on the growth of children and teens during puberty. In addition, global estimates strongly suggest that children do not meet the 2020 World Health Organization (WHO) [3] recommendations for PA, based on which children should engage in PA for at least 60 min daily. Consequently, this makes them excellent candidates for future obesity-related disorders. Promoting PA in children and adolescents benefits a range of medical conditions, including cardiovascular disease, obesity and all-cause mortality, and a range of psychological health problems [4]. A sedentary lifestyle also contributes to decreased academic performance and a delay in cognitive development of children and young people [5]. Furthermore, there is growing empirical evidence of a relationship between a lack of PA and mental health measures. For example, research suggests that overweight teens who do not play sports are more prone to risky behaviors, including suicide attempts and addiction to alcohol and illicit drugs [6].

It has long been known that PA and sport have a positive impact on the physical and mental health of children and young people [7], and systematic reviews and studies have indicated that children and adolescents engaged in increased levels of PA had better physical and mental health and psychosocial well-being than their sedentary peers [7,8].

The most variable component of daily energy expenditure is that due to the PA. Variations in energy expenditure determine body composition, together with energy intake and energy balance, as the energy imbalance is compensated by the mobilization of body fat or its accumulation. Moreover, considerable changes in PA through immobilization or training influence body composition through changes in muscle mass [9].

From a psycho-physiological perspective, acute PA triggers an increase in the neurotransmitters epinephrine and dopamine and brain-derived neurotrophic factors able to enhance cognitive processes [10]. Aerobic exercise can facilitate cognitive functioning by enabling specific aspects of information processing [11]. In fact, the findings of several studies also imply acute exercise can increase the allocation of attention and memory resources and thus benefits executive control function in children, preadolescents, and adolescents [12,13,14].

In this context, the relationship of physical activity and fitness to academic performance is of special interest because physical education programs in schools are required to contribute to the primary mission of schools, i.e., the promotion of academic performance. Current studies have focused on the relationship between PA and the academic performance of school-aged children. A meta-analysis study has demonstrated that, in children, PA participation is associated with better cognitive performance [5]. It should be noted that motor skills [15], cardiorespiratory fitness [16], and cognitive function [17,18,19] are significantly associated with psychological well-being (PWB) [18,19]. PWB is referred to positive mental states, such as happiness or satisfaction, and sometimes this aspect of psychological well-being is referred to as subjective well-being [20]. It has been indicated that PWB includes six parameters: self-acceptance, positive relations with others, autonomy, environmental mastery, purpose in life, and personal growth [21]. Regular participation in physical activities can help children and adolescents to develop their mental health and PWB [22,23]. Therefore, from the point of view of physical and psychological health, providing children with opportunities for PA should be a priority, especially since the prevalence of weight gain is more related to a sedentary lifestyle than to poor eating behavior alone. Unfortunately, children are frequently reluctant to comply with exhortations of healthy lifestyle behaviors; thus, school is considered the preferred environment for promoting PA [24]. The promotion of PA throughout life is a major objective of school-based physical education. However, physical activities during the day have traditionally been less predominant during class time, and the amount of weekly time spent on school-based physical education is limited compared to other subject areas [25]. Since weekly physical activity hours are still well below the recommendations of the World Health Organization (WHO) [3], during the school day, recess would provide regular opportunities for students to move and engage in PA. Several school-based interventions have been able to decrease anthropometric and cardiometabolic risk factors [26], but few interventions have taken into consideration a school program that includes recess at school, an important time for physical recovery and mental health, [27,28] as an important time in establishing, maintaining [24,25], and increasing cognitive functioning, mental health, and well-being [27].

As detailed above, school time is mainly dedicated to the learning of a series of curricular notions with the result of not having enough time to devote to physical education and sport. Thus, here we wanted to investigate the usefulness of an additional method concerning both the performance of physical activity carried out during recess and also for a short time during curricular lessons dedicated to other disciplines. Therefore, the hypothesis that we wanted to test is that a systematic and structured intervention of physical activity conducted daily at school outside the canonical hours set for this can have recognizable and measurable effects on physiology and psychosocial well-being. Consequently, this study was designed in order to describe changes in body composition, physical fitness, cognitive performance, and psychosocial well-being in school children after a 6-month PA intervention consisting of physically active breaks of a duration of 10 min a day, introduced during curricular lessons, together with a 10 min physical activity intervention during the daily school recess period.

## 2. Methods

### 2.1. Participants and Procedures

To calculate the required sample size, G*power was used, thus revealing the need of a total of 288 participants (*n* = 144 per group) to detect a significative effect, with a power of 95%, of the intervention on children’s cognitive performance. To evaluate the effects of promoting PA, we contacted a sample of regular primary schools (in the province of Lecce, Italy) from the network of our research group through personnel contact. We included schools that were willing to participate with a minimum of two classes. Ultimately, 14 classes belonging to 5 different schools located in the three cities were selected. All children in grades 4 and 5 (*n* = 352) were invited to participate. Children and their parents received an informative letter about the study, including an informed consent form. We received the informed consent of 310 children, who were included in the study.

The study was conducted in accordance with the Helsinki Declaration and the European Union recommendations for Good Clinical Practice (document 111/3976/88, July 1990). The University’s Research Ethics Committee approved the study (N.1/2021). Before data collection, all physical education (PE) teachers and research assistants received training on the testing and evaluation protocols.

A sample of 310 children (139 boys vs. 171 girls), aged between 8 and 10 years (9.82 ± 0.51), was selected from schools which in three cities with a similar socioeconomic status and schools that have not previous participated in health promotion programs. The schools were public schools, with attendance from 8:00 to 13:00.

All of the children were healthy and free of any disabilities, musculoskeletal, cardiological, neurological, or respiratory diseases or dysfunctions, or any other conditions limiting their ability to perform exercise and able to abstain from all physical activity outside the parameters of the study protocol during the test days. More specifically, the participants did not undertake new sports competitions or modifications to physical activities already practiced before this study and for those who already practiced sports outside of school, the collaboration of their respective coaches was requested in order not to vary the workload.

**Figure 1:**
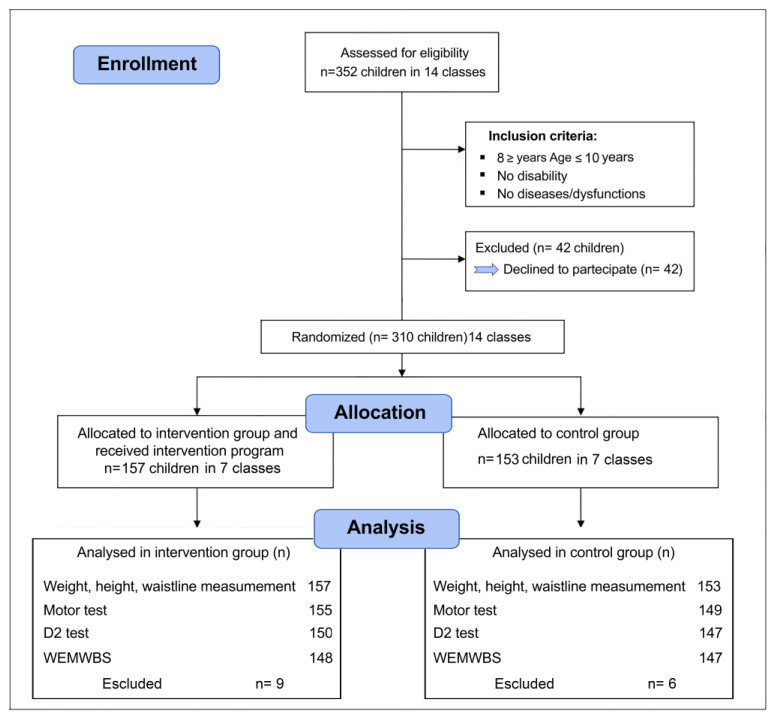
CONSORT flow diagram. WEMWB, Warwick–Edinburgh mental well-being test.

Children were excluded from the data analysis if they did not complete the tests, or did not complete the questionnaire, due to accidents, injuries, or illness. Accordingly, 15 students were excluded from the final data analysis (Figure 1). The randomized controlled trial study design shown in Figure 1 was used; for its execution, we employed two “blind” subjects: the evaluators who carried out the anthropometric measurements and the tests on the one hand and the statistical data analysts on the other.

### 2.2. Randomization

A randomization procedure of the classrooms (a computer-generated list of random numbers using SPSS, version 24) was carried out by the two lead researchers (A.M. and S.M., co-authors). After all of the children and parents completed the baseline assessment, the classrooms were randomized. The baseline assessment consisted mainly of the participants’ anamnesis, i.e., information about surgeries, immunizations, allergies, and illnesses and results of physical exams and tests. Furthermore, information was collected on sports activities, including those of a competitive nature, which were carried out by the participants outside school hours. This evaluation was made through a questionnaire addressed to the parents followed by, only if necessary, a subsequent interview. The basic information required for randomization (initials and participant code, age, weight, and height) was stored in a database. The randomization was stratified by school and grade ensuring that in each school there were both control and intervention classes and that the number of control and intervention classes was balanced between the two grades.

### 2.3. Training Intervention

Before the start of the project, all of the teachers and children in the intervention schools received on-site training to deliver general information to them on the nature and significance of the intervention through three lessons held by a certified physical education teacher. Conversely, the control group did not receive any intervention.

Training intervention involved varied, inclusive, and non-competitive activities, and the school environments of the recruited classes were changed in order to create more opportunities to be physically active during breaks, lessons, and recess. Five min physically active breaks were introduced during Italian, English, mathematics, and science classes during which, alongside theoretical and academic knowledge, the students were asked to carry out various motor tasks. These 5 min active intervals were repeated twice in the morning for a total duration of 10 min a day. It was the teacher who chose when to propose to the class to take an active break: at the beginning of the lesson or between one lesson and another. Specific activity breaks included spelling jacks, walking breaks, jumping jacks, and classroom games. Likewise, during recess, the students were encouraged to engage in structured and semi-structured activities for 10-min. They represent breaks in the school day typically before lunch that involve a variety of planned, inclusive, and actively supervised games and activities. Each activity was designed to improve cardio-respiratory endurance and gross motor skills, as well as the development of different psychomotor abilities, and above all, they were designed to be fun, non-uniform, and repetitive for the children and applicable to schools (Table 1). All of the intervention sessions were facilitated by an activity leader who was a certified PE teacher.

### 2.4. Measures

The study protocol comprised a first evaluation at the start of the study (T0) and a final evaluation after 6 months of intervention (T1). Data were recorded in the gyms in the morning at nine a.m., and the measurements were carried out by the same evaluators in a “blind” mode in all of the schools and in all of the cities. The height of the children was measured with a Seca stadiometer to the nearest 0.1 cm, while their weight was measured with an Omron balance to the nearest 0.5 kg. Body weight and height were ascertained in duplicate with standard techniques. Waist circumference was measured in duplicate at the iliac crest at mid-respiration using a non-elastic measuring tape.

For children and adolescents, the Centers for Disease Control and Prevention defines overweight as a body mass index (BMI: weight in kilograms divided by height in meters squared) between the 85th and 95th percentiles and obesity as a BMI at or above the 95th percentile for sex and age [29].

The ratio between waist circumference and height (WHR) was calculated; in pediatric populations, the cutoff value of 0.48 is used for the WHR to classify abdominal obesity [30].

The relative fat mass (RFM, pediatric) was calculated according to the formula 74—(22 × height (m)/WC (m)) + (5 × sex) using sex = 0 for men and 1 for women; the cut-off points defined by the RFM are more accurate than BMI to provide an estimation of whole-body fat percentage among children [31].

Concerning physical fitness, participants’ levels were determined using a battery of standardized tests. Specifically, they were: the standing long jump test, a test used to assess explosive leg power [32]; the Ruffier test, which is a simple cardiovascular endurance test which involves measuring fitness levels and recovery capacity after physical exertion [33]; and the sit and reach test, which measures the flexibility of the lower back and hamstring muscles [34].

These tests are simple and quick to perform, thereby their use is ideal for a school context. The physical fitness tests were conducted for both the intervention group and control group at baseline (T0) and at the end of the intervention (T1).

Regarding selective attention and concentration, the d2 test of attention was used. The test consists of 14 lines of characters where respondents should cross out d letters with two dots in 20 s. This time limit is allotted for each line separately. Both pre-test and post-test evaluations were performed for each subject and each participant performed the test only once. Performance in the d2 test was assessed using: (i) the total number of responses (TN), including correct answers and errors (E) in the d2 test, a quantitative measure of working speed, (ii) the standardized number of correct answers minus commission errors (CP), an objective measure of concentration, and (iii) the number of all errors (omission error + commission error) relating to the total number of responses (E%), a qualitative measure of accuracy and completeness were used as indictors for evaluating cognitive function. Errors are defined as omission errors (number of correct answers (e.g., “d” with two dashes) missed) and errands of commission (any distracting element such as a “p” or “d” with a dash or more than two incorrectly marked dashes).

Previous research results have indicated that the d2 test has a high degree of reliability to assess cognitive performance in school children to assess the effect of physical activity [12,35,36].

To our knowledge, only a few studies have examined manipulative association PA and cognitive function with PWB in primary school students. The 14-item Warwick–Edinburgh mental well-being scale (WEMWBS) is used to measure students’ PWB, including positive affect (feelings of optimism, cheerfulness, and relaxation) and positive functioning (energy, clear thinking, self-acceptance, personal development, competence, and autonomy) [37]. PE teachers first explained how to fill out the questionnaire to the students. The students were asked to circle the number that best reflected their experience of each statement in the past weeks, using a five-point rating scale. Furthermore, the students were asked to respond to statements such as “I have been feeling optimistic about the future” on a five-point Likert scale (0 = none of the time, 2 = rarely; 3 = some of the time; 4 = often; 5 = all the time).

All 14 items were scored positively and the score of each item was summed to yield the total score of the scale. Previous studies have shown that the WEMWBS is a reliable and valid scale for assessing mental well-being in children [23,38,39]. In this study, the Cronbach’s alpha reliability coefficient of WEMWBS was 0.871, indicating a high degree of internal consistency.

### 2.5. Statistical Analysis

Means and standard deviations were described for the total sample and for both the participant group and the control group.

All of the data were tested for normality using the Kolmogorov–Smirnov test; for normally distributed variables, paired and independent Student’s *t*-tests were used and within-group and between-group differences were evaluated, respectively. A two-way repeated measures ANOVA was used when the subjects had undergone two or more conditions. Effect sizes were calculated as Cohen’s *d* and interpreted as per convention (0.2 = small, 0.5 = medium, and 0.8 = large). Analysis of the qualitative associations between parameters was performed using the Fisher’s exact test.

Statistical significance was accepted as *p* < 0.05.

## 3. Results

The total sample consisted of 310 children (Table 2).

Baseline, follow-up values for anthropometry and fitness variables were collected from the total population as detailed under Methods. Table 2 shows the characteristics of 157 children in the participant group and 153 in the comparison group. Following random assignment of the children to the two groups (intervention and control), there were no significant differences between these groups as regards to the mean values for weight, height, BMI, relative fat mass (RFM), and the proportion of overweight children or children with obesity (*p* > 0.05, by Student’s *t* test; Table 2). Significant differences between the sexes were found only for RFM (*p* < 0.05, by Student’s *t*-test).

**Figure 2:**
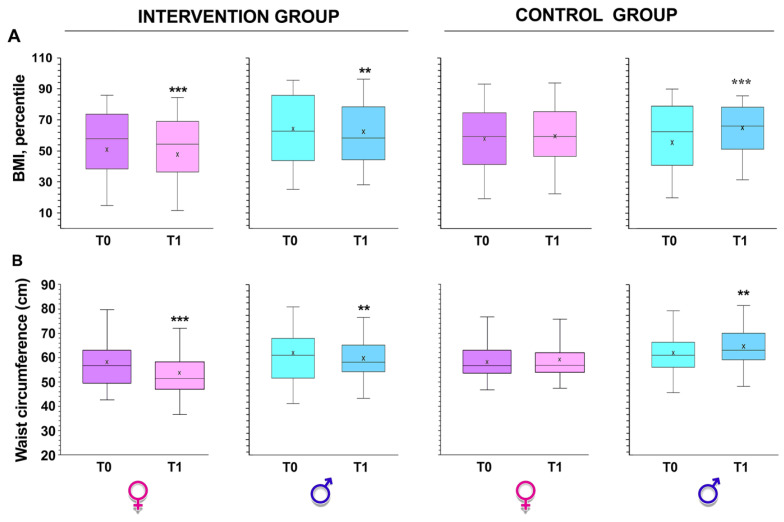
Differences in BMI and waist circumferences between the intervention (**A**) and control (**B**) groups obtained before (T0) and after (T1) intervention. ** *p* < 0.001 and *** *p* < 0.0001 by paired Student’s *t* test.

There was a decrease in total body weight and in BMI in the intervention group compared to the control group (*p* < 0.01 by Student’s *t*-test, Figure 2A). This included a significant increase in BMI points within the boys in the control group during the same period (*p* < 0.001 by Student’s *t*-test, Figure 2A).

Another indicator of obesity is waist circumference; this indicator substantially decreased in the intervention group (*p* < 0.001 from paired Student’s *t*-test; Figure 2B), while it increased significantly in the control group (*p* < 0.0001 by paired Student’s *t*-test; Figure 2B).

Furthermore, in a two-factor repeated measures ANOVA controlling for baseline to T1 scores, there was an overall significant ‘time x group’ difference between the intervention group and the control group for WHR and RFM. The WHR decreased in the intervention group compared to the control group (*F*_1,622_ = 35.68, *p* > 0.99, *η*^2^*_p_* = 0.037, small effect size by Cohen’s *d*; Table 3). We observed a significant difference in “time x group” between the intervention and control groups for both boys (*F*_1,622_ = 35.68, *p* = 0.33, *η*^2^*_p_* = 0.022, small effect size by Cohen’s *d*) and girls (*F*_1,622_ = 35.68, *p* < 0.001, *η*^2^*_p_* = 0.058, small effect size by Cohen’s *d;* Table 3).

Similarly, there was an overall significant ‘time x group’ difference between the intervention group and the control group for RFM (*F*_1,642_ = 14.27, *p* > 0.05, *η*^2^*_p_* = 0.023, small effect size by Cohen’s *d*); this difference was also evident in the boys (*F*_1,642_ = 14.27, *p* = 0.93, *η*^2^*_p_* = 0.021, small effect size by Cohen’s *d*) and in the girls (*F*_1,642_ = 14.27, *p* < 0.05, *η*^2^*_p_* = 0.038, small effect size by Cohen’s *d*; Table 3) groups.

**Figure 3:**
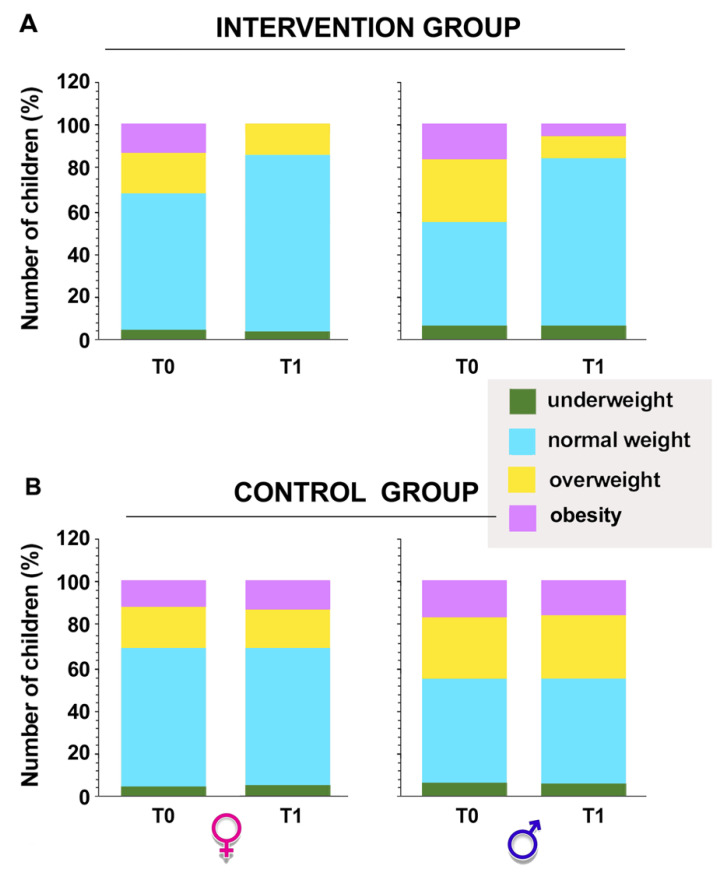
Percentage of overweight, underweight, or normal weight children or children with obesity in the intervention (**A**) and control (**B**) groups obtained pre (T0) and post (T1) intervention. The sample was also divided between girls and boys. Intervention group *p* = 0.0001 by Fisher’s exact test.

On the other hand, significant differences emerged between the proportion of normal weight, underweight, or overweight children or children with obesity between the intervention and control groups and before and after the intervention in the experimental group (*p* = 0.0001 by Fisher’s exact test; Figure 3).

### 3.1. Motor Test Results

**Figure 4:**
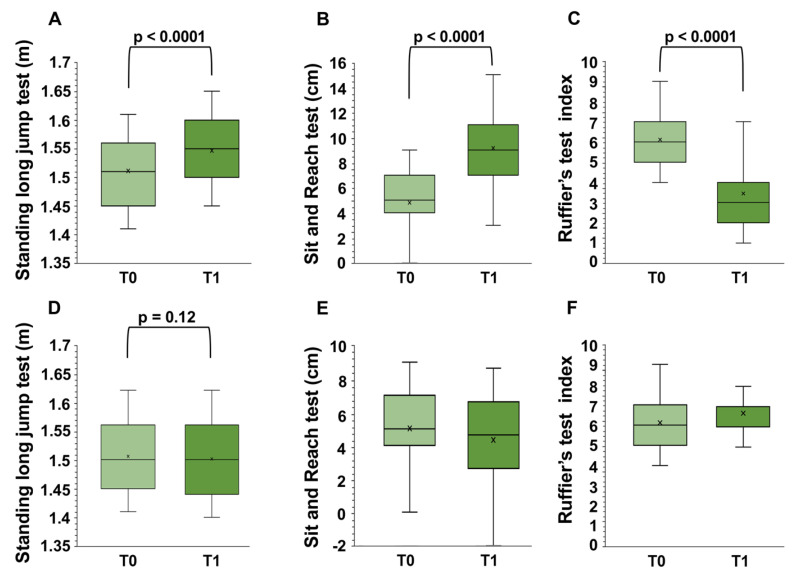
Differences in motor tests in the intervention (**A**–**C**) and control (**D**–**F**) groups before (T0) and after (T1) intervention obtained by two-factor repeated measures ANOVA.

In a two-factor repeated measures ANOVA controlling for baseline to T1 scores, there was a significant ‘time x group’ difference between the intervention group and the control group (Figure 4) in all of the outcome measures. Individual measures for all of the motor tests included the standing long jump test (*F*_1,496_ = 120.56, *p* < 0.001, *η*^2^*_p_* = 0.66, large effect size by Cohen’s *d*), the Ruffier test (*F*_1,496_ = 1442.97, *p* < 0.001, *η*^2^*_p_* = 0.78, large effect size by Cohen’s *d*), and the sit and reach test (*F*_1,28_ = 1558.29, *p* < 0.001, *η*^2^*_p_* = 0.79, large effect size by Cohen’s *d*).

Moreover, post-hoc analysis showed that the intervention group gained a significant increase from T0 to T1 in the standing long jump test (*t* = 18.79, *p* < 0.001, *d* = 1.91 large effect size by Cohen’s *d*), the Ruffier test (*t* = 37.33, *p* < 0.001, *d* = 2.63, large effect size by Cohen’s *d*), and the sit and reach test (*t* = 32.40, *p* < 0.001, *d* = 2.29, large effect size by Cohen’s *d*) scores (Figure 4A–C). The control group did not report significant changes in their standing long jump test and in teach test scores (*p* > 0.05, Figure 4D,F), whilst a decrease in the Ruffier test scores was observed (*p* < 0.001, *d* = 2.06, large effect size by Cohen’s *d*) (Figure 4E). In the intervention group, the effects were the same in the males as in the females.

### 3.2. Cognitive Test

Table 4 shows data on cognitive function (TN, E%, and CP) before (T0) and after intervention (T1) in both groups. The baseline (T0) cognitive test scores did not differ between the girls and boys or between the two groups (Table 4). On the other hand, in T1, significant differences were observed in the two groups regarding the total number of responses (TN, *t* = 36.29, *p* < 0.001, *d* = 1.24, large effect size by Cohen’s *d*) and the concentration performance scores (CP, *t* = 27.12, *p* < 0.01, *d* = 0.6, medium effect size by Cohen’s *d*). Conversely, the number of errors relating to the total number of responses (E%) was greater in the control group than in the intervention group (*t* = 23.32, *p* < 0.01, *d* = 0.6, medium effect size by Cohen’s *d*, Table 4).

The WEMWBS scores ranged from 14 to 70, meaning that the full range of possible values was used. At T0, no significant differences between the two groups (*p* > 0.05) were observed; the mean WEMWBS scores were 48.7 ± 8.6 and 49.2 ± 9.1 for the intervention and control groups, respectively. These high scores for PWB indicate a good level of mental well-being on the basis of the initial population study [37].

**Figure 5:**
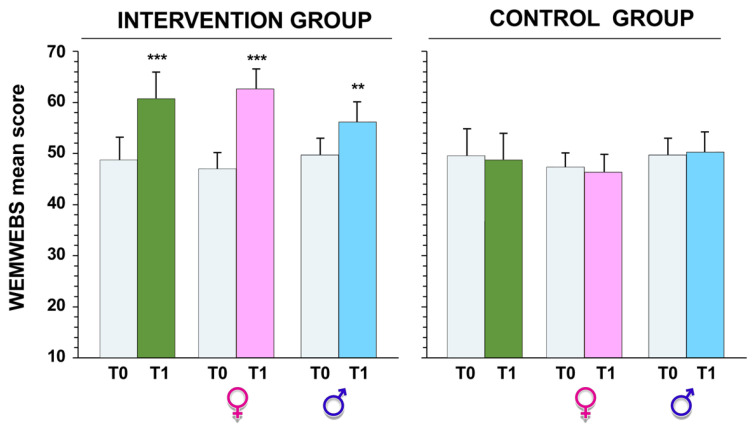
Warwick–Edinburgh mental well-being scale (WEMWBS) score obtained before (T0) and after (T1) intervention, in both the intervention and control groups. ** *p* < 0.01 and *** *p* < 0.001 by paired Student’s *t* test.

Furthermore, we noticed a change in the range of WEMWBS scores from T0 to T1; in the intervention group, the scores ranged from 21 to 70, and the mean WEMWBS score was 58.7 ± 5.7. Thus, well-being levels significantly increased between T0 and T1 (*t* = 26.4, *p* < 0.001, *d* = 2.03 large effect size) regardless of gender (Figure 5).

## 4. Discussion

Obesity in the pediatric population is a serious public health problem, and in southern Europe, there is the highest percentage of overweight or obese children [40,41], with Italy included [1,5,40,41,42,43]. As children spend most of their day in school, they provide the appropriate environment for implementing interventions that accentuate a healthful and active lifestyle [24], for example, by promoting physical activity during breaks or by increasing the number of compulsory hours of physical education classes [44].

This is the first study evaluating the effects of a school-based physical activity intervention, performed in the form of some active breaks of a total duration of 10 min a day, introduced during curricular lessons, together with a 10 min physical activity intervention during the daily school recess period, on obesity prevention, fitness, psychological health, and cognitive performance in Italian school children.

In the intervention group, the school-based physical activity intervention produced the following main consequences: (i) changes in body mass index, in waist circumference, and waist-to-height ratio; (ii) lower relative body fat mass; (iii) better performances in physical fitness; (iv) significant differences in cognitive performance; and (v) enhancement of psychological well-being in the intervention group compared with the control group. Given the conflicting results in the literature regarding physical activity interventions in schools, one of the objectives of this study was to examine the effects of a 6-month obesity prevention and physical activity promotion program in children.

Our results show that the intervention program used here, despite individual variability, produced a decrement in both the mean BMI value and the waistline value in the healthy children of the intervention group compared to the control group. A high percentage of body fat is coupled with cardiovascular risk factors in both children and adolescents [45]. Therefore, given the strong evidence for an association between increased morbidity and body fat in children and adolescents [45,46], it is important to evaluate the possible effectiveness of health interventions on body fat percentage.

The intervention also significantly decreased the WHR in the intervention group. Several studies have reported that the WHR is a very helpful predictor of cardiovascular risk in adults [47,48] and children [49] as well; however, in the latter, it is a better predictor of body fat in boys than in girls [50]. Recently, it was shown that the relative fat mass (RFM) equations modified for pediatric age are superior to BMI and the WHR in predicting whole-body fat percentage in both boys and girls [31]. Here, we noted a benefit in RFM after the intervention.

These findings can mainly be explained by the increase in energy expenditure, as no significant changes in calorie intake were identified during the 6-month period.

Albeit physical-activity-based interventions varying substantially between their intervention approaches, most of them have been demonstrated to have a positive impact on young people [26,51,52]. While not all of the studies showed significant effects on decrementing BMI, nevertheless, all of the studies reported at least one significant improvement in indicator-related health, such as improvements in body fat percentage, sedentary behavior, or serum biomarkers [51]. It is noteworthy to mention that there are also studies that did not report significant enhancements in obesity, BMI, and the WHR, and subsequently the various interventions [52,53], and that in some studies, an adequate control group was missing [54].

The main task of a program for the prevention of childhood obesity is not only to reduce body weight or BMI but, above all, to improve body composition and, consequently, improve motor coordination and physical fitness [55]. Indeed, the results of our study show that school-based intervention significantly improves the physical fitness of children compared to normal physical education. Among others, some of the factors that are often measured for evaluation are cardiorespiratory fitness and muscle strength [56]. Our most important result is the improvement of cardiorespiratory fitness after the intervention, since it is a key marker of health in pediatric age [57,58]. In fact, children with a fit cardiorespiratory system tend to be healthier adults. Thus, good cardiorespiratory fitness correlates to a lower risk of cardiovascular disease, type 2 diabetes, and hypertension at a young age. In addition, children with healthy cardiorespiratory fitness tend to be healthier adults [57,58]. Considering school performance, some studies have reported positive associations between healthy physical conditions and cognitive functioning in children, with cardiorespiratory fitness being the best predictor of such correlations [59]. However, motor control and strength have also been shown to have a positive influence on brain development [59]. Our results obtained with the d2 test show an increase in attention and concentration performance in children in the intervention group compared to those in the control group. Systematic reviews of cross-sectional studies are in agreement with our findings; there are broad, positive associations between physical fitness and cognitive performance in school-aged children [60,61].

The reasons for the improvements in cognitive performance related to exercise are not yet fully understood but there is no doubt that cognitive enhancement is based on different levels of change [61]. Indeed, functional brain changes (cognition-related brain activity patterns) [62] or cellular and molecular changes (variations in plasma lactate concentration, insulin-like growth factor-1 (IGF-1), or brain-derived neurotrophic factor (BDNF)) [63] are associated with exercise-induced improvements in cognitive performance. For example, some current hypotheses consider that the lactate produced by the muscles, crossing the blood–brain barrier, is oxidized to provide energy for cognitive processes [64]. In accordance with other studies [10], we hypothesize that in addition to activation of the neural parts of the brain such as the frontal lobes, participant children are supposed to experience greater excitation of the cerebellum, which is also involved in the mediation of cognitive functions. In addition, as described in previous studies, the development of cardio-respiratory fitness is associated with the development of different brain areas, such as the hippocampus, motor cortex, superior temporal gyrus, and basal ganglia [65]. Since in the pediatric age academic activities are the form of main engagement, and the fact that good cognitive performance could have a considerable impact on academic performance [66], physical activities could help children and adolescents decrease anxiety, improve their mood, and evolve their mental health and PWB [18,19]. In this sense, in the present research, we observed that children who participated in the intervention, not only improved their cognitive functioning, but also their psychological well-being. This is of particular interest, because it has been shown that good cognitive functioning is correlated with better psychosocial adaptation for children and adolescents, better adaptation to the environment, and greater success in several daily activities [18,19]. For the measured variables, we also evaluated the effect of gender; for some of them, we have highlighted small differences in the values of the probabilities returned by the statistical tests, but this does not influence the trend, so that we can assert that the intervention had significant effects on the children regardless of gender.

It is worth remembering that the association between increased physical activity and mental well-being demonstrated here is coincident with several previous literature reports [38] and findings also observed in the context of COVID-19 [67].

Although other unmeasured factors may have contributed to the observed effects (this may be a limitation of the study), the results established that increased physical activity positively contributed to the physical and mental well-being of the participating children, suggesting that physical activity represents a modifiable means to protect well-being by promoting resilience under different conditions. We have to consider that this study was conducted on children aged 8 to 10 years for whom pubertal stages were not measured and the results probably cannot be transferred to the general population of school children with different ages; furthermore, of the participants in this study, we did not assess habitual physical activity. Another limitation could be the sample size and the loss of follow-up data which could compromise the generalizability of the results, because the recruited participants were representative of a target population with considerable individual variability. For example, the influences of socio-economic conditions were not evaluated in the present study. In addition, future studies that take into account variables such as the study time or rest, as well as nutrition or the use of new technologies, which could affect associations, would be recommended. Additionally, only specific physical fitness dimensions have been assessed. Yet another potential limitation of this study is the difficulty of controlling for variations in daily physical activities that were performed by the study participants outside of school hours. However, greater attention was paid by the respective coaches of all of those children who regularly played sports. Future research, therefore, should consider an assessment of different dimensions of fitness (e.g., cardiorespiratory fitness, muscular fitness, and motor-cognitive fitness), which could provide a more comprehensive insight into the relationships between physical fitness, cognitive functioning, and psychological well-being.

## 5. Conclusions

In conclusion, this type of intervention is particularly appreciated by children as it allows curricular activities to be interrupted for a few minutes with physical exercise which is also carried out in the form of a game. These breaks are then associated with the physical activity performed during recess, in order to structure coherent and functional daily physical activity. These results suggest that integrating physical activity allows for a reduction in the BMI of children and increases the levels of physical well-being, thus confirming the evidence of a connections between physical fitness, cognitive functioning, academic performance, and mood improvement. Therefore, schools should create more opportunities for teachers and students to introduce intervention strategies to promote regular exercise, which normally increases fitness levels; facilitating access to physical practice in children can help promote both their health and integral development.

## Figures and Tables

**Table 1:** Active and recess break protocol.

Phase	Type of Exercise	Duration
Warm-up	Walk in place; reach both arms up and down; step side-to-side; press arms forward; roll shoulders forward; alternately lift left and right knees up and down; roll head in a half circle left and right	3′
Training (Main exercise)	Walking Circuits Jump-in and Jump-out Marching down the row Squats Jump rope Chicken dance Dinosaur stomp Geometry dance Carrots being harvested	5′
Cool-down	Yoga poses Reach for the sky; touch toes; arm circles; neck circles; knee to chest; quad stretch; hamstring stretch; breathing deep	2′

**Table 2:** Physical characteristic of the children.

Characteristic Intervention Group	All (*n* = 157)	Boys (*n* = 72)	Girls (*n* = 85)
Age, y	9.9 ± 0.7	10.0 ± 0.4	9.8 ± 0.6
Weight, kg	40.4 ± 10.2	41.6 ± 9.8	39.7 ± 9.4
BMI	18.9 ± 2.6	19.5 ± 3.2	18.6 ± 2.4
BMI, percentile	61.3 ± 11.6	62.3 ± 9.3	58.3 ± 8.3
Waistline	59.1 ± 5.4	62.2 ± 5.2	57.8 ± 3.4
RFM	25.1 ± 1.2	27.33 ± 2.7	25.1 ± 1.2 *
**Control Group**	**All** **(*n* = 153)**	**Boys** **(*n* = 67)**	**Girls** **(*n* = 86)**
Age, y	9.9 ± 0.5	10.0 ± 0.7	9.8 ± 0.7
Weight, kg	40.5 ± 9.9	40.9 ± 10.3	38.7 ± 10.2
BMI	19.0 ± 3.7	19.35± 2.9	19.1 ± 2.4
BMI, percentile	62.5 ± 9.8	62.9 ± 11.6	58.9 ± 10.1
Waistline	58.8 ± 4.6	61.9 ± 5.4	57.7 ± 3.8
RFM	26.2 ± 2.7	25.1 ± 1.6	27.8 ± 2.2 *

Abbreviation: BMI, body mass index (calculated as weight in kilograms divided by height in meters squared). RFM, relative fat mass. * *p* < 0.05 between girls and boys by Student’s *t*-test.

**Table 3:** WHR and RFM measurements at T0 and T1.

		T0			T1		
	All (*n* = 157)	Boys (*n* = 72)	Girls (*n* = 85)	All (*n* = 153)	Boys (*n* = 67)	Girls (*n* = 86)	*p*
**Intervention group**							
WHR	0.44 ± 0.04	0.45 ± 0.01	0.42 ± 0.03	0.43 ± 0.03	0.44 ± 0.01	0.41 ± 0.03	0.0002
RFM	24.9 ± 1.6	25.1 ± 1.3	24.7 ± 1.8	26.4 ± 3.6	27.3 ± 2.7	25.5 ± 4.1	<0.0001
**Control group**							
WHR	0.44 ± 0.01	0.45 ± 0.04	0.42 ± 0.03	0.44 ± 0.04	0.45 ± 0.02	0.42 ± 0.01	>0.05
RFM	24.6 ± 2.7	24.8 ± 2.0	24.6 ± 2.2	25.1 ± 1.8	25.2 ± 2.1	24.8 ± 2.4	>0.05

WHR, waist-to-height ratio; RFM, relative fat mass; *p* significance between T0 and T1 by two-factor repeated measures ANOVA; small effect size by Cohen’s *d*.

**Table 4:** The d2 test performance.

		T0			T1		
	All (*n* = 157)	Boys (*n* = 72)	Girls (*n* = 85)	All (*n* = 153)	Boys (*n* = 67)	Girls (*n* = 86)	*p*
**Intervention group**							
TN	379.35 ± 76	372.75 ± 83	385.75 ± 97	473.21 ± 80	460.43 ± 71	483.76 ± 0.03	
CP	120.75 ± 48	117.13 ± 32	124.87 ± 28	147.88 ± 72	141.32 ± 28	155.52 ± 41	<0.0001
E%	12.1 ± 6	13.76 ± 3	10.4 ± 8	6.7 ± 6	7.9 ± 2	5.5 ± 5	
**Control group**							
TN	379.85 ± 94	375.59 ± 91	383.3 ± 101	392.56 ± 112	388.21 ± 102	397,65 ± 97	
CP	121.67 ± 36	106.35 ± 41	127.27 ± 47	128.63 ± 76	114.56 ± 2.1	130.56 ± 84	>0.05
E%	12.5 ± 6	13.9 ± 7	11.1 ± 8	9.5 ± 7	11.2 ± 8	7.9 ± 3	

TN, total number of responses in the d2 test; CP = concentration performance; E% = percentage of errors; *p* significance between T0 and T1 by two-factor repeated measures ANOVA.

## Data Availability

Data is unavailable due to privacy.
